# Percutaneous Pulmonary Valve Perforation in Pulmonary Atresia With Intact Ventricular Septum: Multicenter Comparison of Radiofrequency Versus Chronic Total Occlusion Wires

**DOI:** 10.1016/j.jscai.2025.104045

**Published:** 2025-11-13

**Authors:** Arash Salavitabar, Sara Conroy, Isaac Kistler, Asaad Beshish, Ryan Callahan, Matthew A. Crystal, Howaida El-Said, Bryan Goldstein, Michael Hainstock, Ray Lowery, Amr Matoq, Daniel McLennan, George T. Nicholson, Brian P. Quinn, Shyam Sathanandam, Jessica Tang, Sara M. Trucco, Wendy Whiteside, Darren P. Berman

**Affiliations:** a The Heart Center, Nationwide Children’s Hospital, Columbus, Ohio; b Biostatistics Resource at Nationwide Children's Hospital, Columbus, Ohio; c Center for Perinatal Research, Nationwide Children’s Hospital, Columbus, Ohio; d Center for Biostatistics, The Ohio State University, Columbus, Ohio; e Emory University School of Medicine, Children’s Healthcare of Atlanta, Atlanta, Georgia; f Children’s Hospital of Philadelphia, Philadelphia, Pennsylvania; g Columbia University Irving Medical Center, New York, New York; h Division of Pediatric Cardiology, Rady Children’s Hospital, San Diego, California; i Heart Institute, UPMC Children’s Hospital of Pittsburgh and Department of Pediatrics, University of Pittsburgh School of Medicine, Pittsburgh, Pennsylvania; j Division of Pediatric Cardiology, University of Virginia Health System, Charlottesville, Virginia; k Division of Pediatric Cardiology, University of Michigan, Ann Arbor, Michigan; l Heart Institute, Cincinnati Children’s Hospital, Cincinnati, Ohio; m Division of Pediatric Cardiology, Children’s Hospital Wisconsin, Milwaukee, Wisconsin; n Department of Paediatric Cardiology, Alder Hey Children’s Hospital, Liverpool, United Kingdom; o Division of Pediatric Cardiology, Monroe Carell Jr. Children's Hospital at Vanderbilt, Nashville, Tennessee; p Department of Cardiology, Boston Children’s Hospital, Boston, Massachusetts; q LeBonheur Children’s Hospital, University of Tennessee, Memphis, Tennessee; r Division of Cardiology, Department of Pediatrics, St. Louis Children’s Hospital, St. Louis, Missouri; s Division of Pediatric Cardiology, Children’s Hospital Los Angeles, Los Angeles, California

**Keywords:** cardiac perforation, chronic total occlusion, pulmonary atresia with intact ventricular septum, pulmonary valve perforation, radiofrequency wire

## Abstract

**Background:**

Percutaneous radiofrequency (RF) wire pulmonary valve perforation (PVP) in pulmonary atresia/intact ventricular septum (PA/IVS) is a high-risk procedure. This study compared procedural efficacy and complications between RF and chronic total occlusion (CTO) wire PVP.

**Methods:**

This multicenter study included patients with PA/IVS who had PVP attempted at 13 centers. Outcomes of interest were efficacy and complications. Univariable logistic regression models were used to estimate odds ratios and risk differences.

**Results:**

PVP was attempted in 206 patients; median age 3.0 days (1.0-6.0) and weight 3.1 kg (2.7-3.5). The RF PVP was attempted first in 165 (80.0%) patients, CTO in 27 (13.1%), and stiff end of the coronary wire in 14 (6.8%). The procedural success rate of 94% was similar between wire types. CTO group utilized smaller initial valvuloplasty balloons (2.25 mm [2-3] vs 4 mm [3-6]) and >2 balloons in 71% of cases (vs 18% in RF). Overall and major procedural complications occurred in 26.5% versus 12.5% (odds ratios [OR], 2.5 [0.8-11.1]; *P* = .15) and 14.8% vs 4.2% (OR, 4.0 [0.8-73.2]; *P* = .18), respectively, of RF versus CTO groups. Major postprocedural complications occurred in 12% versus 4.2% of the RF versus the CTO groups. Cases with multiple (vs single) PVP attempts had higher overall (40.4% vs 17.0%; OR, 3.3 [1.7-6.6]; *P* < .01) and major (24.6% vs 8.5%; OR, 3.5 [1.5-8.3], *P* < .01) procedural complication rates.

**Conclusions:**

PVP remains an effective treatment strategy in PA/IVS. Complication rate differences between CTO and RF wires may be clinically relevant. Further studies with larger cohorts are warranted to confirm findings and guide optimal intervention strategies.

## Introduction

**Figure 1:**
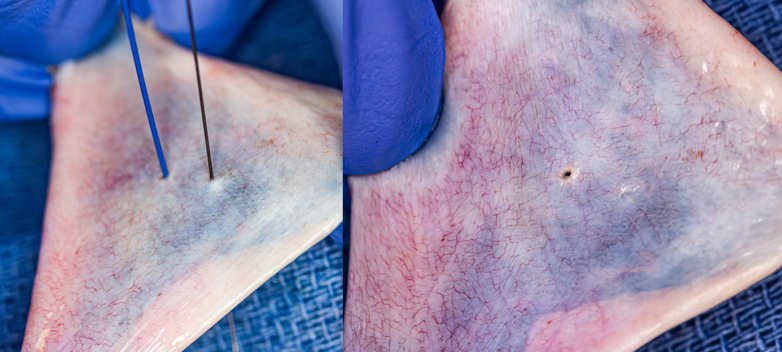
**Perforation of pericardial tissue using radiofrequency wire (blue) and 0.014” wire (black)**. The picture on the right shows persistence of a hole from the radiofrequency perforation, but no visible hole using a 0.014” wire.

Pulmonary atresia with intact ventricular septum (PA/IVS) presents with significant anatomical variability that ultimately results in physiology ranging from single-to-biventricular circulation. Candidacy for right ventricular (RV) decompression in these patients depends on perceived adequacy of the RV size and morphology, tricuspid valve (TV) Z-score, presence or absence of RV-dependent coronary circulation (RVDCC), and pulmonary valve (PV) morphology. Patients who are thought to be candidates for “1.5 ventricle” or biventricular physiology and are amenable to RV decompression can be treated successfully with percutaneous PV perforation (PVP) and balloon pulmonary valvuloplasty (BPV). Radiofrequency (RF) wire PVP is the most commonly utilized technique for creation of RV-to-PA continuity in this procedure;^1^, ^2^, ^3^, ^4^, ^5^, ^6^, ^7^, ^8^, ^9^, ^10^, ^11^, ^12^, ^13^, ^14^, ^15^, ^16^, ^17^, ^18^, ^19^, ^20^, ^21^ however, the use of chronic total occlusion (CTO) wires^22^, ^23^, ^24^, ^25^, ^26^, ^27^, ^28^ and older studies showing the use of stiff ends of coronary wires^8^^,^^11^^,^^14^^,^^29^, ^30^, ^31^, ^32^, ^33^ have been described. Although RF, CTO, and coronary wire PVP have been shown to be effective tools for PVP in small studies, this procedure remains high-risk, and complications can be catastrophic.^34^^,^^35^ The mechanism of RF wire PVP is to cause a “burn” lesion, which naturally results in a perforation larger than the wire diameter with potential to cause greater hemodynamic compromise with inadvertent cardiac or PA perforation. This compares with a 0.014” CTO or coronary wire, which causes a smaller perforation (Figure 1).

The aims of this study were (1) to compare procedural efficacy and complication rates between attempted PVP in patients with PA/IVS with CTO versus RF wires and (2) to determine patient and procedural risk factors associated with adverse events with both CTO and RF PVP. Our hypotheses were that (1) CTO wire PVP in patients with PA/IVS has fewer major complications compared with RF perforation and that (2) the procedural efficacy of CTO wire PVP is not worse than that of RF PVP.

## Materials and methods

### Study design and patient selection

This was a retrospective, observational multicenter study including all patients with PA/IVS who had attempted percutaneous wire PVP from 2007-2019 at 13 centers. Patient identification was performed with a combination of the Congenital Cardiac Catheterization Project on Outcomes (C3PO) collaborative database and institutional databases. A PVP attempt was defined as wire advancement with the intent to perforate the PV, as determined by the operator. Patients were excluded if a cardiac catheterization was performed, but PVP was not attempted, and if pulmonary atresia was present with an alternative baseline anatomy (eg, tetralogy of Fallot, ventricular septal defect). Patients with any degree of antegrade pulmonary blood flow were excluded. Data were collected on demographic characteristics, echocardiographic and angiographic data, procedural data, and adverse events. In addition to RF and CTO wires, the use of the stiff end of coronary wires was included to capture crossover to RF and CTO. As this study was observational in nature, wire type was determined at the discretion of the individual providers.

Outcomes of interest were procedural efficacy and complications. Procedural efficacy was defined as successful percutaneous creation of a RV-to-PA connection before leaving the catheterization lab. The presence of RVDCC was determined at the discretion of the individual providers. Procedural complications were further separated into a composite variable for major complications, which included RV outflow tract/infundibular perforation, pulmonary artery perforation, TV injury, pericardial effusion requiring emergent drain placement, cardiac arrest, need for extracorporeal membrane oxygenation (ECMO) support, stroke, emergent surgery, or death. A composite variable was utilized due to difficulty in retrospectively deciphering each major complication’s etiology, of which there could be multiple sequelae. Postprocedural complications during the index hospitalization also formed a composite variable for major complications, which included pericardial effusion requiring drain placement, cardiac arrest, need for ECMO, seizure, stroke, or death. The potential for misclassification bias of complications was addressed by utilizing data from a combination of the adjudicated C3PO database and institutional chart review.

### Statistical analysis

Demographic characteristics, clinical, and procedural characteristics were summarized using count and percentage for categorical variables and median (IQR) for continuous variables. The initial wire type stratified all descriptive tables for patient characteristics. Descriptions and tables summarizing complications excluded patients who had wire type crossover in order to capture complications representative of individual wire types. Missed data in demographic characteristic and descriptive information was described in table footnotes. Complete case analyses were used for primary and secondary aims.

The hypotheses were addressed using univariable logistic regression models to estimate odds ratios and risk difference with 95% CI for overall and major complications, as well as for procedural efficacy between wire types. Additional analyses compared complications for infants with only 1 attempt to those with more than 1 attempt, regardless of wire type. No adjustments were made for potential confounders or center effects due to the small sample size and low event rate. As a sensitivity analysis to assess the impact of low counts on the logistic regression models, logistic regression models for the hypotheses were also fit using exact logistic regression. Following best statistical practice and STROBE reporting guidelines, *P* values are not presented in descriptive tables.^36^, ^37^, ^38^ The *P* values are interpreted on a continuum with smaller *P* values supporting evidence that the data are incompatible with the null hypothesis.^39^ All analyses were performed using R version 4.2.2.

### Ethical statement

This research was carried out in accordance with the appropriate ethical guidelines. This study was approved by each institutional review board and was shared using study-specific data use agreements, with data protected to avoid ethical issues. This study was approved by the C3PO collaborative.

## Results

### Patient characteristics and anatomic features

Patient characteristics are described in Table 1, and anatomic features are described in Table 2. PVP was attempted in 206 patients at a median age of 3.0 days (IQR, 1.0-6.0) and a weight of 3.1 kg (2.7-3.5). Membranous atresia was noted in 191 (96%) patients, the median PV annulus z-score was −1.9 (−2.5 to −1.2), the median TV annulus z-score was −1.1 (−1.9 to 0.1), and the most common RV morphology was tripartite (n = 100, 75.2%). RVDCC was present in 1 patient (0.5%). Three (1.5%) patients had a cardiac intervention prior to PVP attempt, including a fetal intervention attempt at PV dilation (n = 1), surgical aortopulmonary shunt placement (n = 1), and a prior unsuccessful PVP attempt at an outside institution prior to transfer for the index PVP attempt at a study site (n = 1).

**Table 1:** Patient characteristics by wire type of first attempt.

Characteristic	Overall (N = 206)	RF (n = 165)	CTO (n = 27)	Coronary wire (n = 14)
Age at catheterization, d^a^	3.0 (1.0-6.0)	3.0 (1.0-6.0)	2.0 (1.5-4.0)	4.0 (2.3-5.8)
Gestational age, wk^b^	38.0 (37.0-39.0)	38.1 (37.0-39.0)	38.0 (36.0-39.0)	36.0 (33.0-38.2)
Weight at catheterization, kg	3.1 (2.7-3.5)	3.2 (2.8-3.6)	3.2 (2.8-3.4)	2.4 (1.9-3.2)
BSA at catheterization, m^2^^c^	0.2 (0.2-0.2)	0.2 (0.2-0.2)	0.2 (0.2-0.2)	0.2 (0.1-0.2)
Male sex	109 (52.9%)	85 (51.5%)	15 (55.6%)	9 (64.3%)
Genetic syndrome/abnormality^d^	15 (7.5%)	13 (8.1%)	2 (7.4%)	0 (0.0%)
Extra-cardiac abnormalities^e^	24 (11.8%)	19 (11.6%)	4 (14.8%)	1 (7.7%)
Prenatal diagnosis	104 (50.5%)	81 (49.1%)	17 (63.0%)	6 (42.9%)
Previous cardiac interventions	3 (1.5%)	2 (1.2%)	1 (3.7%)	0 (0.0%)

Values are median (IQR) or n (%).

BSA, body surface area; CTO, chronic total occlusion; RF, radio frequency.

a One patient missing age at catheterization; in RF.

b Six patients missing GA; all RF.

c Seventeen patients missing BSA; 14 RF, 2 CTO, 1 coronary.

d Five patients missing data on genetic syndrome / abnormality; 4 RF, 1 coronary.

e Two patients missing data on extra-cardiac abnormalities; 1 RF, 1 coronary.

**Table 2:** Baseline cardiac features by wire type of first attempt.

Characteristic	Overall (N = 206)	RF (n = 165)	CTO (n = 27)	Coronary wire (n = 14)
PV morphology^a^				
Membranous atresia	191 (96.0%)	155 (96.3%)	25 (100.0%)	11 (84.6%)
Muscular atresia	8 (4.0%)	6 (3.7%)	0 (0.0%)	2 (15.4%)
PV annulus, mm^b^	5.9 (5.0-6.8)	6.0 (5.0-6.9)	5.8 (5.0-6.5)	5.5 (5.0-6.7)
PV annulus (z-score)^c^	−1.9 (−2.5 to −1.2)	−1.9 (−2.5 to −1.2)	−2.2 (−2.5 to −1.7)	−1.6 (−2.0 to −0.8)
TV annulus (mm)^d^	9.0 (7.3-11.0)	9.1 (7.5-11.0)	8.0 (6.6-10.8)	8.5 (7.9-10.4)
TV annulus (z-score)^e^	−1.1 (−1.9 to 0.1)	−1.0 (−1.8 to 0.1)	−0.7 (−2.2 to 0.6)	−1.2 (−1.6 to 0.0)
Tricuspid regurgitation^f^				
None/trivial	11 (5.4%)	7 (4.3%)	4 (15.4%)	0 (0.0%)
Mild	38 (18.8%)	33 (20.4%)	2 (7.7%)	3 (21.4%)
Mild-moderate	33 (16.3%)	29 (17.9%)	2 (7.7%)	2 (14.3%)
Moderate	87 (43.1%)	65 (40.1%)	15 (57.7%)	7 (50.0%)
Moderate-severe	18 (8.9%)	16 (9.9%)	1 (3.8%)	1 (7.1%)
Severe	15 (7.4%)	12 (7.4%)	2 (7.7%)	1 (7.1%)
RV morphology^g^				
Unipartite	2 (1.5%)	2 (1.9%)	0 (0.0%)	0 (0.0%)
Bipartite	31 (23.3%)	25 (23.6%)	3 (18.8%)	3 (27.3%)
Tripartite	100 (75.2%)	79 (74.5%)	13 (81.3%)	8 (72.7%)
RV-CA connections^h^				
No connection	158 (80.2%)	127 (79.9%)	18 (72.0%)	13 (100.0%)
Yes, RV-dependent	1 (0.5%)	0 (0.0%)	1 (4.0%)	0 (0.0%)
Yes, RV non-dependent	38 (19.3%)	32 (20.1%)	6 (24.0%)	0 (0.0%)

Values are median (IQR) or n (%).

CA, coronary artery; CTO, chronic total occlusion; PV, pulmonary valve; RF, radiofrequency; RV, right ventricle; TV, triscuspid valve.

a Seven missing data on PV morphology; 4 RF, 2 CTO, 1 coronary.

b Twelve missing PV annulus; 9 RF, 3 CTO.

c Twenty-two missing PV annulus z-score; 18 RF, 4 CTO.

d Sixteen missing TV annulus; 10 RF, 6 CTO.

e Twenty missing TV annulus z-score; 14 RF, 6 CTO.

f Four missing data on tricuspid regurgitation; 3 RF, 1 CTO.

g Seventy-three missing data on RV morphology; 59 RF, 11 CTO, 3 coronary.

h Seven missing data on presence of RV-CA connections; 4 RF, 2 CTO, 1 coronary; 2 with RV-CA connection missing data on dependence (both RF).

### Procedural characteristics and outcomes

**Figure 2:**
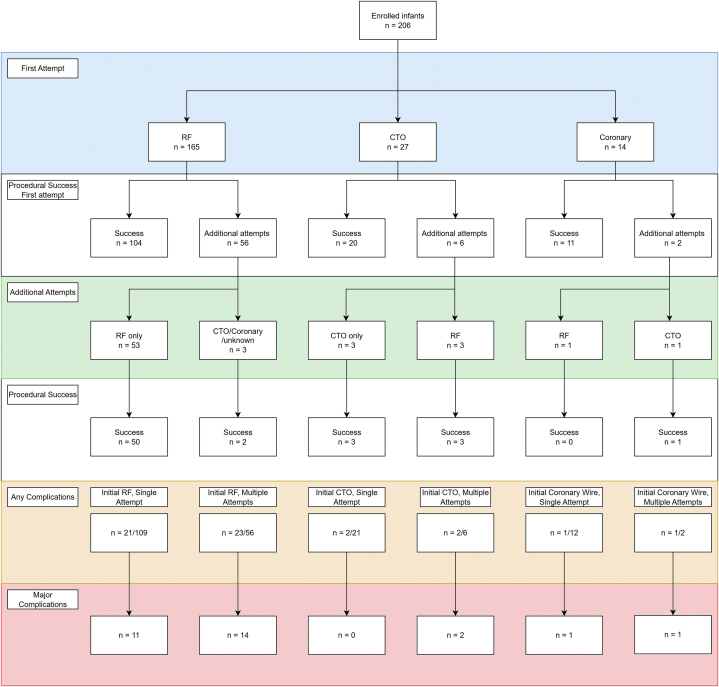
**Flowchart of enrolled patients and outcomes, separated by wire choice on first attempt.** CTO, chronic total occlusion; RF, radiofrequency.

Of the 206 PVPs, RF perforation was attempted first in 165 (80.1%) patients, CTO in 27 (13.1%), and the stiff end of the coronary wire in 14 (6.8%). Figure 2 displays the method of PVP, the number of attempts, and the crossover between wire types. Overall, there was a rare crossover between wire types, including 2 patients from the Coronary Wire group, one each to RF and CTO. Most patients had 1 PVP attempt (n = 141, 68.4%), with up to 8 attempts reported in 1 patient (Supplemental Table S1). Institutional variation in wire type use is shown in Supplemental Figure S1. A successful PVP was reported on the first attempt in 20 (74.1%) patients in the CTO wire group and 104 (63.0%) in the RF group. The overall PVP procedural success rate was 94.2%. When excluding wire type crossover, the PVP success rate was 95% with RF and 96% with CTO (odds ratios [OR], 0.8 [95% CI, 0.0-4.8], *P* = .86). The 2 patients with unipartite RVs had unsuccessful PVPs, without major complications.

The most common vascular access was a femoral vein (n = 192, 93.2%) (Supplemental Table S1). The procedure duration was a median of 122 minutes (90.5-164.0) in CTO versus 103.0 (71.0-142.0) in RF. Transcatheter coronary evaluation was performed in 186/205 (90.7%) patients. The most common CTO wires utilized were the Asahi Confianza (Asahi Intecc) (n = 11, 32%), followed by Asahi Astato XS 20 (n = 5, 15%), and the most common tip load was 20 grams (n = 5, 42%), with most being unrecorded.

**Figure 3:**
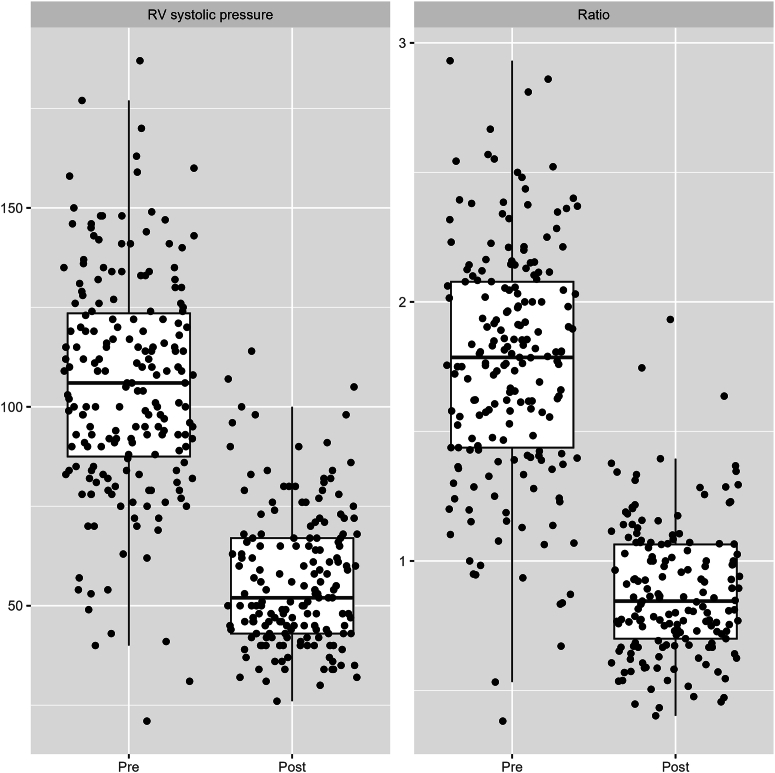
**Comparison of right ventricular (RV) systolic pressure in mm Hg (left) and right ventricular-to-systemic pressure ratio (right) pre and post-PVP and balloon pulmonary valvuloplasty.**

Following PVP, BPV was performed with a median initial balloon diameter of 4 mm (3, 6) in the RF group and 2.25 mm (2, 3) in the CTO group (Supplemental Table S2). The CTO group used >2 balloons for BPV in 71% cases, compared with 18% in the RF group. There were improvements in RV systolic pressure (median 105 mm Hg [87-124] to 52 mm Hg [43-67], *P* < .001) and RV/systemic systolic pressure ratio (median 1.8 [1.44-2.08] to 0.8 [0.69-1.05], *P* < .001) following PVP and BPV (Figure 3). Patent ductus arteriosus stent implantation was attempted or performed during 15% (n = 31) of index catheterizations.

### Intraprocedural complications

Across all wire types, there was a 23.7% overall intraprocedural complication rate with a 13.1% rate of major intraprocedural complications (Table 3). There was a higher rate of overall (26.5% vs 12.5%, OR, 2.5 [0.8-11.1], *P* = .15) and major (14.8% vs 4.2%, OR, 4.0 [0.8-73.2], *P* = .18) complications in the RF versus CTO groups (Table 4). However, poor precision of the confidence intervals indicated model instability resulting from low cell counts. The results from the sensitivity analyses using exact logistic regression were largely similar, but with a slight increase in uncertainty. The difference in complications was similar when combining the CTO and Coronary Wire groups, and the imprecision of the confidence intervals remained. Major complications comprised 55% of all complications (Supplemental Table S3). The rate of cardiac or PA perforation was 10.6% (n = 21) (n = 20 [12.3%] in RF vs n = 1 [4.2%] in CTO) (Table 3). The 1 CTO PVP that resulted in a pericardial effusion was managed with emergent drain placement, whereas major complications among the RF group were comprised of 5 (11.6%) cardiac arrests, 14 (32.6%) pericardial drains, 6 (14.0%) emergent surgeries, 1 (2.3%) need for ECMO, and 1 (2.3%) death (Table 3).

**Table 3:** Intraprocedural complications between wire type groups.

Characteristic	Overall (N = 198)	RF (n = 162)	CTO (n = 24)	Coronary wire (n = 12)
Any complication	47 (23.7%)	43 (26.5%)	3 (12.5%)	1 (8.3%)
Any major complication	26 (13.1%)	24 (14.8%)	1 (4.2%)	1 (8.3%)
RVOT/infundibular perforation	7 (3.5%)	6 (3.7%)	1 (4.2%)	0 (0.0%)
Pulmonary artery perforation	12 (6.1%)	12 (7.4%)	0 (0.0%)	0 (0.0%)
Perforation—other	2 (1.0%)	2 (1.2%)	0 (0.0%)	0 (0.0%)
Tricuspid valve injury	1 (0.5%)	1 (0.6%)	0 (0.0%)	0 (0.0%)
Cardiac arrest	6 (3.0%)	5 (3.1%)	0 (0.0%)	1 (8.3%)
Pericardial effusion requiring emergent drain placement	16 (8.1%)	14 (8.6%)	1 (4.2%)	1 (8.3%)
ECMO	2 (1.0%)	1 (0.6%)	0 (0.0%)	1 (8.3%)
Stroke	1 (0.5%)	1 (0.6%)	0 (0.0%)	0 (0.0%)
Death	2 (1.0%)	1 (0.6%)	0 (0.0%)	1 (8.3%)
Emergent surgery	7 (3.5%)	6 (3.7%)	0 (0.0%)	1 (8.3%)
Other major complication	1 (0.5%)	1 (0.6%)	0 (0.0%)	0 (0.0%)
Any minor complication	26 (13.1%)	24 (14.8%)	2 (8.3%)	0 (0.0%)
Vascular occlusion	4 (2.0%)	4 (2.5%)	0 (0.0%)	0 (0.0%)
Arrhythmia requiring intervention	15 (7.6%)	14 (8.6%)	1 (4.2%)	0 (0.0%)
Other minor complication	7 (3.5%)	6 (3.7%)	1 (4.2%)	0 (0.0%)

ECMO, extracorporeal membrane oxygenation; RVOT, right ventricular outflow tract.

**Table 4:** Complications comparing radiofrequency (RF) to chronic total occlusion (CTO) wire type, excluding patients who switched wire types after initial attempt.

Outcome	Overall	RF	CTO	Risk difference (%) (95% CI)	Odds ratio (95% CI)	*P* value
Any complication	46/186 (24.7%)	43/162 (26.5%)	3/24 (12.5%)	14.0 (−0.8 to 28.9)	2.5 (0.8-11.1)	.15
Major complication	25/186 (13.4%)	24/162 (14.8%)	1/24 (4.2%)	10.6 (1.0-20.3)	4.0 (0.8-73.2)	.18

Values are n/N (%)

When compared to those with 1 PVP attempt, cases with more than 1 PVP attempt had a higher rate of overall (40.4% vs 17.0%; OR, 3.3 [1.7-6.6], *P* < .01) and major (24.6% vs 8.5%; OR, 3.5 [1.5-8.3], *P* < .01) complications, whereas procedural success was similar (94.6% vs 95.0%, *P* = .91) (Table 5). There were no differences in median PV annulus diameters (5.9 mm [4.7-6.8] vs 5.9 mm [5.0-6.9]); OR, 0.95 [0.7-1.2], *P* = .7) and PV Z-scores (−2.2 [−3.2 to −1.5] vs −1.8 [−2.5 to −1.1]; OR, 0.7 [0.48-1.0], *P* = .06) between patients with and without a major complication.

**Table 5:** Complications and success comparing a single PVP attempt to those with more than one PVP attempt.

Outcome	Overall	1 Attempt	>1 Attempt	Risk difference (%) (95% CI)	Odds ratio (95% CI)	*P* value
Any complication	47/198 (23.7%)	24/141 (17.0%)	23/57 (40.4%)	23.3 (9.2-37.5)	3.3 (1.7-6.6) *P* < .01	<.01
Major complication	26/198 (13.1%)	12/141 (8.5%)	14/57 (24.6%)	16.1 (4.0-28.1)	3.5 (1.5-8.3) *P* < .01	<.01
Success	187/197 (94.9%)	134/141 (95.0%)	53/56 (94.6%)	−0.4 (−7.3 to 6.5)	0.9 (0.2-4.4) *P* = .91	.91

Values are n/N (%)

Of the 12 patients who ultimately did not have procedural success, 10 (83%) had major complications resulting in an aborted procedure. Of the 5 patients who had a single RF wire PVP attempt and had an unsuccessful technical result, 4 (80%) had a major complication. Of the 4 patients who started the procedure with an RF attempt and had multiple subsequent attempts (with either RF wire or 1 unknown wire type), all 4 (100%) had major complications. One patient who underwent an unsuccessful CTO wire PVP attempt had no complications. One patient who underwent an unsuccessful coronary wire attempt had a major complication. One patient who underwent an initial PVP attempt with a coronary wire, followed by an attempt with a RF wire, had a major complication.

### Post-PVP course

The overall median hospital length of stay was 19 days (IQR, 12-30). On discharge, transthoracic echocardiography, TR was ≤ mild in 104 (54%) patients, which was improved from the 49 (24%) preintervention. PR was at least moderate in 91 (50%) patients at discharge. RV systolic function was normal or mildly depressed in 147 (83%) patients on discharge, improved from 88 (47%) preintervention.

During the index hospitalization, postprocedural complications were reported in 48 (24%) patients, with major complications in 22 (11%) (Supplemental Table S4). The incidence of any major postprocedural complication was 12% (n = 20) in the RF group and 4.2% (n = 1) in the CTO group. Of the 26 patients with a major intraprocedural complication, 11 (42%) had a major postprocedural complication, including 1 (4%) postprocedural death. The 1 patient with noted RVDCC who underwent successful PVP did not have an intraprocedural complication but did have postprocedural low cardiac output and troponin leak consistent with coronary ischemia, with resultant cardiac arrest.

## Discussion

PA/IVS is a heterogeneous disease that requires a thoughtful, collaborative approach. Neonatal interventions for PA/IVS are high-risk.^34^^,^^35^ Percutaneous PVP provides an important initial nonsurgical option in patients who have adequate tricuspid valves, a patent infundibulum, and an absence of RVDCC. This procedure often involves technical complexities and requires measures of risk mitigation. The choice of wire with which to perform PVP has important implications on both the potential efficacy and safety of this high-risk procedure. This multicenter study provides a comparison of procedural outcomes between RF and CTO wire PVP, demonstrating similar rates of successful RV decompression. The authors found the magnitude of the difference in rate of major complications between RF and CTO wire PVP to be of potential clinical importance in this cohort (Central Illustration), although confidence intervals were wide and included the null value.

**Central Illustration:**
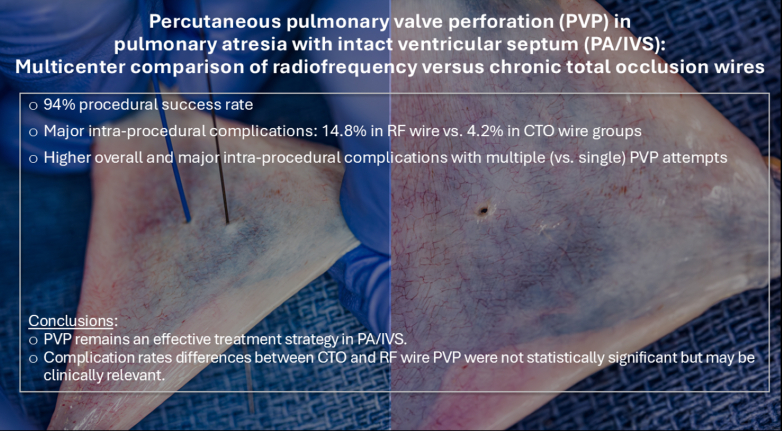
**This multicenter study comparing radiofrequency versus chronic total occlusion wire pulmonary valve perforation provides clinically relevant procedural information about this high-risk procedure.** CTO, chronic total occlusion.

Similar to prior reports of PVP attempts, tripartite RV morphology and normal tricuspid valve Z-scores were most common in our patients.^2^, ^3^, ^4^^,^^8^^,^^11^^,^^12^^,^^15^^,^^20^ The 95% PVP success rate in our study is similar to or better than previously published rates of 75% to 100%,^2^, ^3^, ^4^^,^^7^^,^^11^^,^^12^^,^^14^^,^^15^^,^^17^^,^^19^^,^^20^^,^^22^, ^23^, ^24^^,^^26^^,^^27^ with similarly effective improvement in RV pressure and RV/systolic pressure ratio after PVP and BPV.^2^^,^^3^^,^^7^^,^^14^, ^15^, ^16^^,^^20^ The smaller initial balloon diameter and greater number of balloons needed to perform effective BPV in the CTO group are likely explained by the fact that a smaller hole is created during perforation, which is also the innate advantage of CTO wires in terms of safety profile.

Inadvertent cardiac and/or vascular perforation, most commonly of the RV outflow tract or MPA, remains a concern with this procedure and with potentially catastrophic sequelae.^1^, ^2^, ^3^, ^4^, ^5^^,^^7^^,^^8^^,^^10^, ^11^, ^12^^,^^14^^,^^17^, ^18^, ^19^, ^20^^,^^23^^,^^27^ Our study showed a major complication rate of 13.1%. The major complication rates between the RF versus CTO wire groups were 14.8% and 4.2%, respectively. Although we consider this clinically relevant in this cohort, the difference between these rates had wide confidence intervals, and the generalizability of these results may be limited. Only 1 patient in the CTO group experienced a pericardial effusion that was managed with pericardial drain placement. Although it is reasonable to believe that other patients who received CTO PVP had inadvertent perforation of surrounding structures, the inherent advantage of the wire is demonstrated in Figure 1. Removing a 0.014” wire tip from an inadvertent perforation likely results in a spontaneous seal in most instances, compared with a burned, more permanent hole with RF energy. This fundamental difference is our proposed explanation for the clinically relevant difference in major complications observed in this study and described in smaller case series.^22^^,^^23^ Petit et al^18^ reported on the use of primarily RF wires for RV decompression and, when excluding patients with antegrade pulmonary blood flow, found a 15.4% incidence of RV outflow tract or MPA perforation. Factors associated with cardiac perforation were greater RF energy and longer duration of RF energy in that study. There is an innate additional risk provided by each PVP attempt in a given patient, which was demonstrated in our study by the strong association between multiple PVP attempts and major complications. The need for multiple PVP attempts can also represent challenging patient anatomy and/or technical factors that cannot be accounted for by standard anatomic categories and annular dimensions but can result in greater potential for major complications. Additionally, multiple PVP attempts were more likely in the RF group, which may have confounded the major complication rate comparison between multiple and single PVP attempts. Given the limited power, additional subanalyzes were not performed of outcomes of single versus multiple PVP attempts by each wire type.

In this study, the periprocedural mortality rate was 2% (n = 4). This is lower than studies reporting a 3% to 21% mortality rate with primarily RF wire PVP^2^, ^3^, ^4^^,^^7^^,^^14^ and comparable with those studies reporting 0% to 4% mortality rate where primarily CTO wire or variable wire type was used.^10^^,^^11^^,^^16^^,^^18^, ^19^, ^20^^,^^22^^,^^24^^,^^26^ There were no mortalities in the CTO wire group in our study.

The RVDCC is a known risk factor for major adverse events due to the known sequelae of coronary ischemia with RV decompression in that setting.^11^^,^^23^^,^^40^ The 1 patient in our study who had documented RVDCC with successful PVP went on to develop coronary ischemia and low cardiac output, resulting in a cardiac arrest and need for mechanical circulatory support. RVDCC remains an important contraindication to RV decompression in PA/IVS, regardless of the ability to perform successful PVP.

### Limitations

It is likely that the retrospective patient identification in this study did not identify some patients with attempted, but unsuccessful, PVP. Given that procedures were performed by various operators across multiple centers, the approach to attempted PVP could not be standardized. Furthermore, operator experience and specific technique-associated risk factors were not available for analysis. PA/IVS remains a relatively rare congenital heart defect, which makes analysis difficult to power. This limited power likely affected our ability to estimate the difference in major complications between RF and CTO wire PVP with high precision and did not allow us to statistically account for anatomic, technical, and center-specific associations with outcomes. There was also institutional variation in both case volume and wire type utilized for PVP that could not be controlled for by the center. The decision to perform PVP, versus other strategies, was also institution-specific and not standardized for this retrospective study, and thus, we only included patients who were felt by each institution to be a candidate for PVP. The lack of ability to control for confounders and center-specific effects limits the ability to determine causality as well as generalizability. The CTO wire manufacturer and tip load were difficult to correlate with procedural outcomes in this study, as there was a great variety in wire choice, a lack of information on wire characteristics, and a limited number of complications in the CTO group. Given the small number of those patients, it is difficult to determine anatomic or procedural factors associated with the need for conversion between wire types.

## Conclusion

Percutaneous PVP remains an important and effective treatment strategy in PA/IVS. The success rate of CTO wire PVP was similar to that of RF wire PVP in our study. There was no evidence of difference in major complications between CTO and RF wire PVP, and although the comparison of complication rates can be considered clinically relevant, generalizability and ability to determine causality are limited. Multiple PVP attempts had greater odds of major complications, compared with a single attempt. Further studies with larger cohorts and greater power are warranted to confirm these findings and guide optimal intervention strategies.
