# Supplementary material for: Percutaneous Pulmonary Valve Perforation in Pulmonary Atresia With Intact Ventricular Septum: Multicenter Comparison of Radiofrequency Versus Chronic Total Occlusion Wires
Source: J Soc Cardiovasc Angiogr Interv. 2025 Nov 13;4(12):104045. doi: 10.1016/j.jscai.2025.104045 (PMC12766049; doi:10.1016/j.jscai.2025.104045)

**Supplemental Table 1.** Procedural characteristics by wire type of first attempt

| **Characteristic** | **Overall**  n = 206 | **RF**  n = 165 | **CTO**  n = 27 | **Coronary wire**  n = 14 |
| --- | --- | --- | --- | --- |
| Total number of PV perforation attempts |  |  |  |  |
| 1 | 141 (68.4%) | 108 (65.5%) | 21 (77.8%) | 12 (85.7%) |
| 2 | 35 (17.0%) | 30 (18.2%) | 4 (14.8%) | 1 (7.1%) |
| 3 | 19 (9.2%) | 17 (10.3%) | 1 (3.7%) | 1 (7.1%) |
| 4 | 5 (2.4%) | 5 (3.0%) | 0 (0.0%) | 0 (0.0%) |
| 5 | 5 (2.4%) | 4 (2.4%) | 1 (3.7%) | 0 (0.0%) |
| 8 | 1 (0.5%) | 1 (0.6%) | 0 (0.0%) | 0 (0.0%) |
| Procedure duration (min)^1^ | 105.5 (75.5, 142.0) | 103.0 (71.0, 142.0) | 122.0 (90.5, 164.0) | 102.5 (89.3, 128.3) |
| Fluoroscopy time (min)^2^ | 34.8 (26.0, 52.5) | 34.0 (26.0, 50.2) | 48.0 (31.0, 95.4) | 38.3 (26.7, 44.1) |
| Contrast volume (cc)^3^ | 18.0 (13.1, 25.5) | 18.0 (13.0, 24.5) | 25.5 (16.5, 33.8) | 15.0 (6.5, 16.9) |
| Radiation dose (mGy)^4^ | 167.0 (81.3, 264.3) | 181.0 (92.8, 283.6) | 166.0 (73.0, 268.5) | 90.4 (54.3, 176.3) |
| Radiation DAP (uGy*m2)^5^ | 445.0 (160.0, 1,048.6) | 431.5 (140.4, 1,072.6) | 590.4 (323.5, 994.0) | 615.5 (220.5, 2,504.5) |
| Vascular Access for first attempt |  |  |  |  |
| Femoral artery | 8 (3.9%) | 7 (4.2%) | 0 (0.0%) | 1 (7.1%) |
| Femoral vein | 192 (93.2%) | 153 (92.7%) | 26 (96.3%) | 13 (92.9%) |
| Multiple | 4 (1.9%) | 4 (2.4%) | 0 (0.0%) | 0 (0.0%) |
| Right internal jugular | 1 (0.5%) | 0 (0.0%) | 1 (3.7%) | 0 (0.0%) |
| Umbilical Vein | 1 (0.5%) | 1 (0.6%) | 0 (0.0%) | 0 (0.0%) |
| PDA stent attempted / performed | 31 (15.0%) | 20 (12.1%) | 9 (33.3%) | 2 (14.3%) |
| Coronary evaluation performed^6^ |  |  |  |  |
| No | 19 (9.3%) | 13 (7.9%) | 5 (18.5%) | 1 (7.1%) |
| Yes, during previous catheterization | 8 (3.9%) | 6 (3.7%) | 1 (3.7%) | 1 (7.1%) |
| Yes, during this catheterization | 178 (86.8%) | 145 (88.4%) | 21 (77.8%) | 12 (85.7%) |
| Success for first attempt | 135 (65.5%) | 104 (63.0%) | 20 (74.1%) | 11 (78.6%) |
| RF – Radio Frequency; CTO – Chronic Total Occlusion; PV – Pulmonary Valve; DAP – Dose Area Product; PDA – Patent Ductus Arteriosus | | | | |
| ^1^10 patients missing procedure duration; 8 RF, 2 coronary | | | | |
| ^2^6 patients missing fluoroscopy time; all RF | | | | |
| ^3^8 patients missing contrast volume; all RF | | | | |
| ^4^52 patients missing radiation dose; 49 RF, 3 CTO | | | | |
| ^5^67 patients missing radiation DAP 61 RF, 3, CTO, 3 coronary | | | | |
| ^6^1 patient missing data on coronary evaluation; in RF | | | | |

**Supplemental Table 2.** Balloon characteristics used for balloon pulmonary valvuloplasty, excluding wire type crossover.

| **Characteristic** | **Overall**, N = 198^1^ | **RF**, N = 162^1^ | **CTO**, N = 24^1^ | **Coronary**, N = 12^1^ |
| --- | --- | --- | --- | --- |
| Initial balloon diameter (mm) | 4.0 (3.0, 6.0) | 4.0 (3.0, 6.0) | 2.25 (2.0, 3.0) | 3.75 (3.0, 6.0) |
| Unknown | 10 | 9 | 1 | 0 |
| Number of balloons used |  |  |  |  |
| 0 | 3 (1.6%) | 3 (1.9%) | 0 (0%) | 0 (0%) |
| 1 | 54 (28%) | 50 (32%) | 0 (0%) | 4 (33%) |
| 2 | 83 (43%) | 74 (47%) | 6 (26%) | 3 (25%) |
| 3 | 33 (17%) | 22 (14%) | 9 (39%) | 2 (17%) |
| 4 | 15 (7.9%) | 6 (3.8%) | 7 (30%) | 2 (17%) |
| 5 | 3 (1.6%) | 1 (0.6%) | 1 (4.3%) | 1 (8.3%) |
| Unknown | 7 | 6 | 1 | 0 |
| Largest balloon diameter (mm) | 7.0 (6.0, 8.0) | 7.0 (6.0, 8.0) | 7.0 (7.0, 8.0) | 7.0 (6.0, 8.0) |
| Unknown | 10 | 9 | 1 | 0 |

^1^Median (IQR); n (%)

**Supplemental Table 3:** Complication rates among those patients with complications present, excluding wire type crossover.

| **Characteristic** | **Overall**  N = 47^1^ | **RF**  N = 43^1^ | **CTO**  N = 3^1^ | **Coronary wire**  N = 1^1^ |
| --- | --- | --- | --- | --- |
| Any major complication | 26 (55.3%) | 24 (55.8%) | 1 (33.3%) | 1 (100.0%) |
| RVOT / Infundibular perforation | 7 (14.9%) | 6 (14.0%) | 1 (33.3%) | 0 (0.0%) |
| Pulmonary artery perforation | 12 (25.5%) | 12 (27.9%) | 0 (0.0%) | 0 (0.0%) |
| Perforation - other | 2 (4.3%) | 2 (4.7%) | 0 (0.0%) | 0 (0.0%) |
| Tricuspid valve injury | 1 (2.1%) | 1 (2.3%) | 0 (0.0%) | 0 (0.0%) |
| Cardiac arrest | 6 (12.8%) | 5 (11.6%) | 0 (0.0%) | 1 (100.0%) |
| Pericardial effusion requiring emergent drain placement | 16 (34.0%) | 14 (32.6%) | 1 (33.3%) | 1 (100.0%) |
| ECMO | 2 (4.3%) | 1 (2.3%) | 0 (0.0%) | 1 (100.0%) |
| Stroke | 1 (2.1%) | 1 (2.3%) | 0 (0.0%) | 0 (0.0%) |
| Death | 2 (4.3%) | 1 (2.3%) | 0 (0.0%) | 1 (100.0%) |
| Emergent Surgery | 7 (14.9%) | 6 (14.0%) | 0 (0.0%) | 1 (100.0%) |
| Other major complication | 1 (2.1%) | 1 (2.3%) | 0 (0.0%) | 0 (0.0%) |
| Any minor complication | 26 (55.3%) | 24 (55.8%) | 2 (66.7%) | 0 (0.0%) |
| Vascular Occlusion | 4 (8.5%) | 4 (9.3%) | 0 (0.0%) | 0 (0.0%) |
| Arrhythmia requiring intervention | 15 (31.9%) | 14 (32.6%) | 1 (33.3%) | 0 (0.0%) |
| Other minor complication | 7 (14.9%) | 6 (14.0%) | 1 (33.3%) | 0 (0.0%) |
| RVOT – Right Ventricular Outflow Tract | | | | |

**Supplemental Table 4.** Individual level post-procedural complications between wire type groups, excluding wire type crossover.

| **Characteristic** | **Overall**, N = 198^1^ | **RF**, N = 162^1^ | **CTO**, N = 24^1^ | **Coronary**, N = 12^1^ |
| --- | --- | --- | --- | --- |
| Any complication | 48 (24%) | 40 (25%) | 5 (21%) | 3 (25%) |
| Major complications | 22 (11%) | 20 (12%) | 1 (4.2%) | 1 (8.3%) |
| Cardiac arrest | 9 (4.5%) | 8 (4.9%) | 1 (4.2%) | 0 (0%) |
| ECMO | 2 (1.0%) | 2 (1.2%) | 0 (0%) | 0 (0%) |
| Seizure | 3 (1.5%) | 3 (1.9%) | 0 (0%) | 0 (0%) |
| Stroke | 4 (2.0%) | 4 (2.5%) | 0 (0%) | 0 (0%) |
| Death | 2 (1.0%) | 2 (1.2%) | 0 (0%) | 0 (0%) |
| Other | 12 (6.1%) | 11 (6.8%) | 0 (0%) | 1 (8.3%) |
| Minor complications | 33 (17%) | 26 (16%) | 5 (21%) | 2 (17%) |
| Vascular occlusion | 18 (9.1%) | 12 (7.4%) | 4 (17%) | 2 (17%) |
| Other | 19 (9.6%) | 17 (10%) | 2 (8.3%) | 0 (0%) |
| ^1^n (%) | | | | |

**Supplemental Figure 1.** Institutional variation in wire type use for cases, excluding wire type crossover.


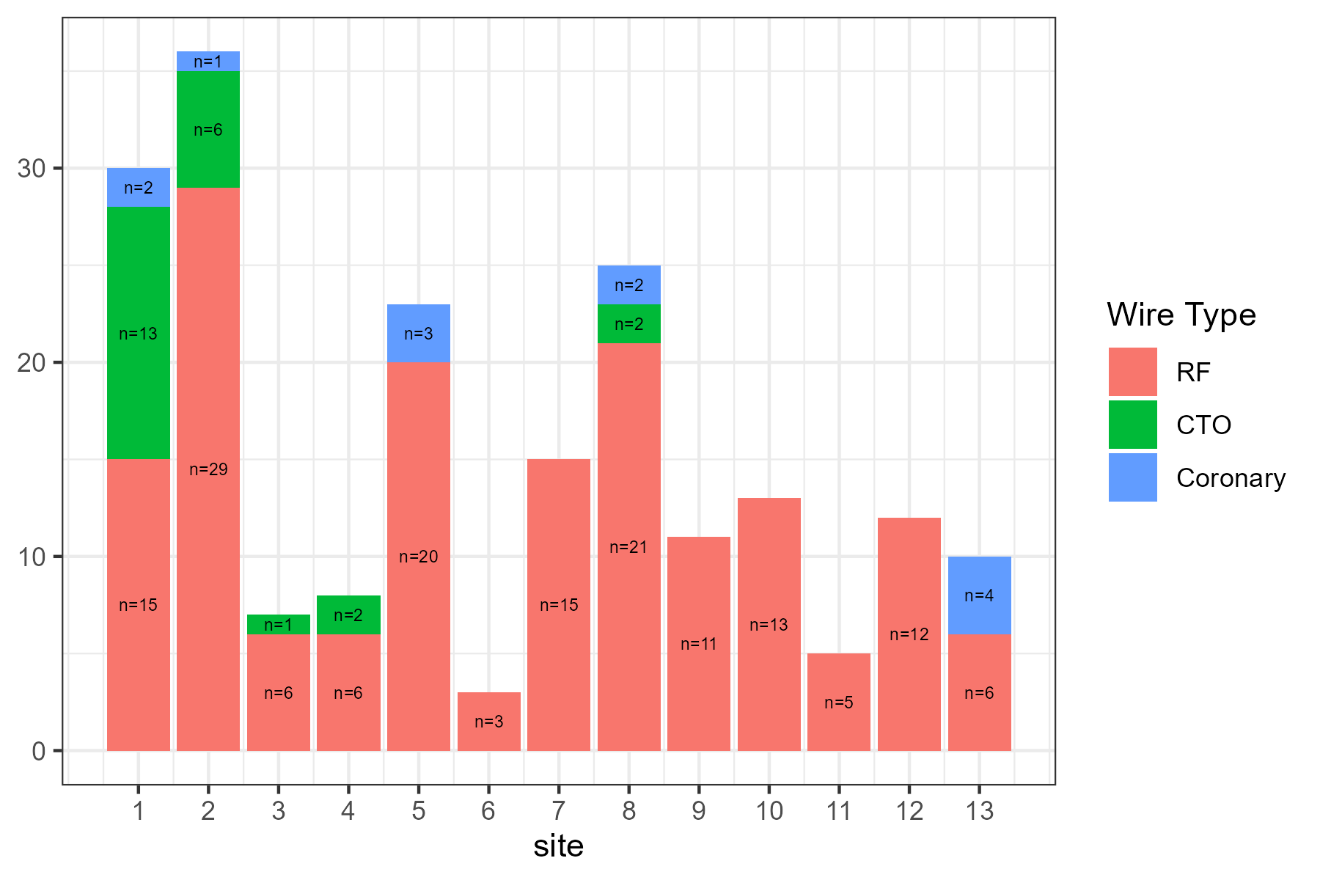

Supplement: Supplementary material [file mmc1.docx]
